# Editorial: Molecular insight of chronic infections

**DOI:** 10.3389/fmicb.2022.1112456

**Published:** 2023-01-04

**Authors:** Honghua Hu, Leyi Wang, Qi Zhao

**Affiliations:** ^1^Jinhua Institute of Zhejiang University, Jinhua, Zhejiang, China; ^2^Faculty of Medicine, Health and Human Sciences, Macquarie University, Sydney, NSW, Australia; ^3^Veterinary Diagnostic Laboratory, Department of Veterinary Clinical Medicine, College of Veterinary Medicine, University of Illinois, Urbana, IL, United States; ^4^School of Computer Science and Software Engineering, University of Science and Technology Liaoning, Anshan, Liaoning, China

**Keywords:** chronic infections, metagenomics, meta-transcriptomics, metabolome, resistome, pathogenesis, protein-protein interactions

Chronic infections are prolonged infections not cleared by treatment and become persistent and latent, causing mobility issues and mortality in patients. To date, there is still no drug to tackle chronic infections efficiently in clinical settings. This Research Topic aims to better understand the molecular basis of chronic infections in both host and pathogen, which will facilitate early intervention and targeted therapeutic strategies.

With advanced technologies, microbiome studies have moved from 16S rRNA gene studies to complete genome (metagenomics), transcriptome sequencing (meta-transcriptomics), and metabolome and resistome characterization. This Research Topic collects new insights, the latest discoveries to understand the molecular insight and pathogenesis of chronic infections, and advanced methods to better predict and study chronic infections. We have received 10 submissions, six of which were published in this Research Topic, including two original research papers on chronic obstructive pulmonary disease (COPD), one original research paper on chronic periodontitis, one review paper on the pathogenesis of inflammatory bowel syndrome (IBD), and two papers using advanced bioinformatics methods in predicting IBD and protein-protein interactions (PPIs) in chronic infections, respectively.

COPD is the third-leading cause of death worldwide, causing 3.23 million deaths in 2019 (WHO newsroom, 2022). Yang et al. explored the changes in the abundance of microorganisms and the different expression levels of host genes between acute exacerbation COPD (AECOPD) and stable COPD using metagenomic and meta-transcriptomic approaches. This study found that there was no significant difference in sputum transcriptionally active microbiome at every level between AECOPD and stable COPD, but the expression levels of five host genes significantly increased in stable COPD. The five differentially expressed host genes play a part in immune response and inflammatory pathways and may provide a clue to investigate the mechanism of COPD and potential biomarkers in the clinical diagnosis and treatment of COPD.

Antibiotics alter the gut microbiome and cause dysbiosis, which leads to antibiotic-resistant organisms. Cho et al. investigated the association between the distribution of antibiotic resistance genes (ARGs), bacterial composition, and antibiotic treatment patterns in patients with COPD and *Clostridioides difficile* infections who had chronic or acute intermittent use of antibiotics and compared them with healthy individuals. This study demonstrated the enrichment of ARGs in antibiotic-affected gut microbiomes and correlated the abundance of resistomes with antibiotic use and gut bacterial diversity.

Periodontitis is the inflammation of periodontal tissue that results from the host's immune response to bacterial infection and can cause alveolar bone resorption (Marchesan et al., 2020). Wei et al. investigated the salivary microbial community and metabolic characteristics in patients with generalized periodontitis and revealed the distinct differences in the salivary microbiome and metabolomics between periodontitis and healthy controls. Striking differences were observed in the composition of salivary metabolites between the aggressive periodontitis and chronic periodontitis groups. It is possible to understand the potential process underlying periodontitis by the integration of microbial and metabolomic data, which may also provide useful biomarkers to monitor the incidence and progress of periodontitis.

IBD is a chronic inflammatory condition of the gastrointestinal tract (Ng et al., 2017). Crohn's disease (CD) and ulcerative colitis (UC) are the two major clinical forms of IBD. Zhang et al. reviewed bacterial species associated with human inflammatory bowel disease and their pathogenic mechanisms and proposed a three-stage pathogenesis model to illustrate the roles of different IBD-associated bacterial species and gut commensal bacteria in the development of human IBD. The current treatment strategy for IBD is to use anti-inflammatory and immune-suppressive drugs. Given that microbes in the gastrointestinal tract, including both the gut commensal and the initiating pathogenic microbes, are the drivers of inflammation in these stages, the authors recommend microbe-targeted therapeutic strategies. The authors proposed to reduce gut microbe loads or eliminate triggering microbes in lesion areas to end the immune responses and restore the intestinal barrier. This may be best managed by locally delivering antimicrobial agents targeting the lesion areas.

Liñares-Blanco et al. generated a microbiome signature with the predictive capacity to identify inflammatory bowel disease (IBD) from fecal samples using a publicly available database and machine learning models. The predictive capacity was validated in two independent external cohorts containing CD and UC, respectively. Their results demonstrated the strong relationship between the intestinal microbiome and IBD. They also showed that these two subtypes have similar microbial patterns and found genera that play an important and common role in their development.

Prediction of protein-protein interactions (PPIs) is crucial for understanding the molecular basis of biological processes, such as chronic infections. Hu et al. established an efficient Network-based Graphical Probabilistic Model (NGPM) for PPI prediction by integrating Gene Ontology (GO) information about proteins, thus alleviating the negative influence of noise data. They proposed a novel scoring function by combining the membership distributions of proteins with network paths. Experimental results show that NGPM has a promising performance and reveal that the consideration of modularity in PPI networks provides a more flexible way to accurately predict PPIs.

Finally, we thank all the authors who contributed their original work to our Research Topic and the reviewers for their valuable comments. We would like to express our sincere gratitude to the editorial office of Frontiers in Microbiology, for their excellent support and for providing us with this opportunity to hold this Research Topic successfully.

## Author contributions

HH and QZ drafted the manuscript. LW revised it. All authors made a direct and intellectual contribution to the work and approved the final version for publication.
